# Effects of El Niño-Southern Oscillation on human visceral leishmaniasis in the Brazilian State of Mato Grosso do Sul

**DOI:** 10.1590/0074-02760190298

**Published:** 2020-02-27

**Authors:** Antonio Brandão da Silva, Everton Falcão de Oliveira, César Claudio Cáceres Encina, Helen Rezende de Figueiredo, Antonio Conceição Paranhos, Alessandra Gutierrez de Oliveira

**Affiliations:** 1 Universidade Federal de Mato Grosso do Sul, Programa de Pós-Graduação em Doenças Infecciosas e Parasitárias, Faculdade de Medicina, Campo Grande, MS, Brasil; 2 Universidade Federal de Mato Grosso do Sul, Instituto de Biociências, Laboratório de Parasitologia Humana, Campo Grande, MS, Brasil; 3 Universidade Federal de Mato Grosso do Sul, Laboratório de Geoprocessamento para Aplicações Ambientais, Faculdade de Engenharias, Arquitetura e Urbanismo e Geografia, Campo Grande, MS, Brasil

**Keywords:** visceral leishmaniasis, neglected diseases, spatial analysis, climate changes

## Abstract

**BACKGROUND:**

Leishmaniases are considered a major public health problem in South America, specifically in Brazil. Moreover, the transmission and epidemiology of leishmaniasis are possibly associated with climatic and environmental variations.

**OBJECTIVE:**

This study aimed to assess the association between the extreme climatic phenomenon El Niño-Southern Oscillation (ENSO), the maximum and minimum variations of temperature, precipitation, and soil moisture and the incidence of visceral leishmaniasis (VL) in Mato Grosso do Sul (MS), Brazil, from 2002 to 2015.

**METHODS:**

The Niño 3.4 index was used for the ENSO variation. The other climatic data were obtained from the climatic tool TerraClimate. Records regarding VL were obtained from the Notification of Injury Information System.

**FINDINGS:**

From 2002 to 2015, there were 3,137 cases of VL recorded in MS. The annual incidence of the disease was negatively associated with the ENSO index and soil moisture in MS. The VL incidence increased during the negative phase of ENSO and decreased during the positive phase.

**MAIN CONCLUSIONS:**

The results demonstrated that the interannual cycles of incidence of human VL are significantly associated with the occurrence of the ENSO phenomenon and its phases, El Niño and La Niña.

Approximately 350 million people are at risk of *Leishmania* spp. protozoan infection, the second most common disease behind malaria that affects a significant number of people annually. Visceral leishmaniasis (VL) is considered the most severe clinical form of leishmaniases with a mortality rate of 90% if left untreated, specifically in children, elderly, and immunosuppressed patients. *Leishmania* parasites are transmitted through the bites of infected female sandflies.^1^

According to the World Health Organization, a total of 200,000-400,000 new cases of VL are reported, accounting for approximately 20,000 deaths annually.^1^ In South America, approximately 95% of VL was recorded in Brazil. In 2013, the country registered more than a thousand human VL cases.^2^

The protozoan *Leishmania infantum*, an etiological agent of VL, is transmitted by *Lutzomyia longipalpis* and *Lu. cruzi*. The mode of transmission of *L. infantum* is considered important to significantly understand the components of the epidemiological chain. In Brazil, VL might present different epidemiological profiles considering that the climatic characteristics, phytophysiognomic mosaics, and socioeconomic conditions of each region significantly vary.^3^ Sandfly vectors are influenced by environmental variables. Temperature, rain, and moisture are the three major variables that influence sandflies’ biology. Higher than expected rainfall reduces the amount of the available nutrients for immature sandflies in the soil and increases soil moisture, limiting larvae and pupae development.^4^ ^,^ ^5^ On the contrary, higher temperatures and lower humidity levels lengthen the adult sandflies’ lifespan and promote female oviposition.^6^

Climate change possibly results in extreme weather events, which have been associated with the outbreaks of various infectious and parasitic diseases worldwide, such as cholera, malaria, dengue, and leishmaniasis.^7^ Considering these extreme climatic events, the *El Niño-*Southern Oscillation (ENSO) phenomenon, which consists of three phases, *El Niño*, *La Niña*, and neutral phase, should be emphasised. These phases are observed as a result of the anomalies in the sea surface temperature (SST) of the Equatorial Pacific Ocean, which has a significant influence on the climate of the Americas.^8^ The Pacific Ocean comprises the largest water mass on the planet; therefore, any variation in its temperature has a direct impact on the climate in different regions on Earth and can modify rainfall and temperature regimes. Thus, ENSO may result in an above-average rainfall in one region, while it may cause extreme drought in another region, in addition to temperature anomalies, with cooler and warmer periods than normal.^8^ ^,^ ^9^

Although climate changes are considered global phenomena, their effects vary according to region, and regional studies assessing the association between the epidemiology of VL and climate change are required in clinical practice. In MS, the transmission of VL has already been associated with different socioeconomic conditions;^10^ however, the association between climate change and the occurrence of VL has not been determined yet. Therefore, this is the first study conducted in Midwest Brazil that aimed to analyse the association between the extreme climatic phenomenon ENSO, the maximum and minimum variations of temperature, precipitation, and soil moisture and the incidence of VL.

## MATERIALS AND METHODS

**Fig. 1:**
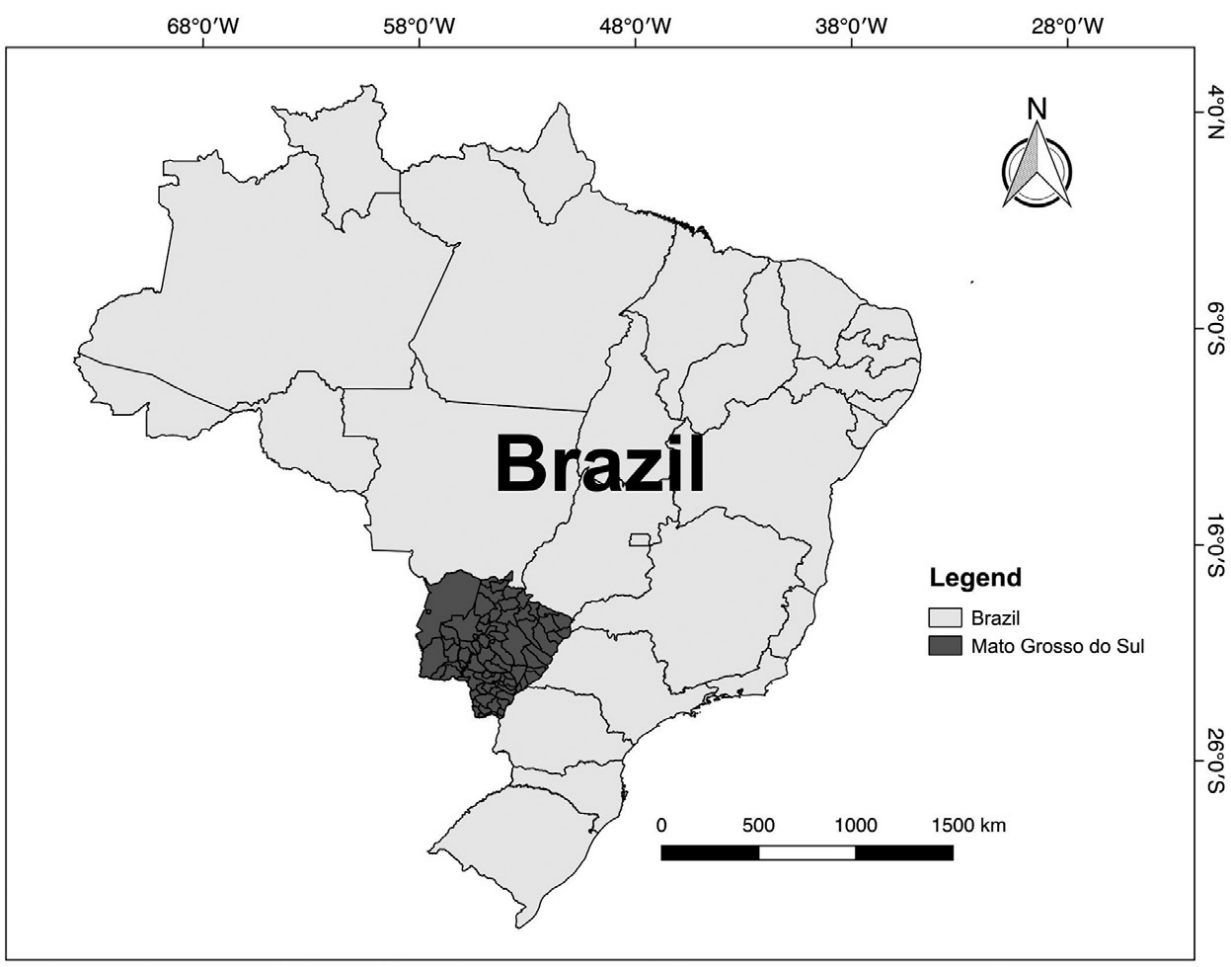
localisation of the study area, Mato Grosso do Sul, in the Midwest Region of Brazil. Source: Brazilian Institute of Geography and Statistics (IBGE), modified.

*Study area* - The State of Mato Grosso do Sul (MS) has an area of 357,145,534 km^2^. It is located in the midwest region of Brazil and is bordered by Paraguay and Bolivia and the states of Paraná, São Paulo, Minas Gerais, Goiás, and Mato Grosso. The state comprises 79 municipalities, with Campo Grande as its capital city (Fig. 1). The state has an estimated 2,682,386 inhabitants, and according to the demographic census of 2010, 85.6% of the population live in an urban area.

According to the Köppen climate classification, there are three types of climate in MS - Aw (wet tropical climate with rainy season in summer and dry season in winter), Cfa (warm temperate climate with hot summer), and Cwa (humid temperate climate with dry winter and hot summer); the last one is restricted to the eastern end of the state. The average annual temperature and rainfall are approximately 25ºC and 1,500 mm, respectively. The majority of the vegetation of the state is composed of Cerrado *lato sensu* (tropical savanna-like climate); however, Pantanal (wetland-like climate) areas in the west and Atlantic Forest areas in the south and southeast have been reported. The hydrographic basin comprises the Paraná and Paraguay rivers.^11^

*Climatic data* - We used the ENSO 3.4 index to assess the climatic phenomena. ENSO is calculated based on SST anomalies in the *Niño* 3.4 region, which comprises the latitude 5ºN-5ºS and longitude 120º-170ºW, obtained from the latest version of the Extended Reconstructed Sea Surface Temperature version 4 sensor. ENSO data were obtained from the online platform available through the Climate Prediction Center, linked to the National Oceanic and Atmospheric Administration of the United States. Positive and negative variations are recorded from the average temperature history in the Equatorial Pacific. Anomalies less than -0.5ºC or greater than 0.5ºC for at least five consecutive months are considered cold phase/*La Niña* or warm phase/*El Niño*, respectively, whereas values in that interval (-0.5, 0.5) are considered the normal/neutral phase.^8^

The maximum temperature, minimum temperature, precipitation, and soil moisture data were obtained from the high-resolution dataset TerraClimate.^12^ Using the free software program QGIS^®^, MS areas were divided, and the average values were obtained monthly for each year (2002-2015).^13^

*Human visceral leishmaniasis cases* - The records of VL cases from each MS municipality were obtained from the Notification of Grievance Information System through the online platform of the Health Information System (TABNET-DATASUS) from 2002 to 2015. These data are in the public domain and are under the responsibility of the Ministry of Health.

*Statistical analysis* - First, the dataset was characterised by the descriptive statistics of the variables of interest. The annual crude incidence of VL (per 100,000 inhabitants) was calculated for the state and municipalities with autochthonous reports during the study. Regarding the cumulative crude incidence of the whole period, 2002-2015, we considered the population in 2008 since this year represents half of the period under analysis.

The Shapiro-Wilk test was used to verify the assumption of the normal distribution of the variables.

Correlation analyses were used to confirm the association between the incidence of VL and the independent variables (ENSO, maximum and minimum temperature, precipitation, and soil moisture). The Pearson correlation coefficient was used when the variables presented normal distribution and linear behaviour. In the absence of one of these characteristics, the Spearman correlation coefficient was used. To obtain an overview of the association between the variables under study, a dispersion and correlation matrix was constructed.

The level of significance was 5% (α = 0.05). The analyses were performed using the software R version 3.4.0.^14^

## RESULTS

**Fig. 2:**
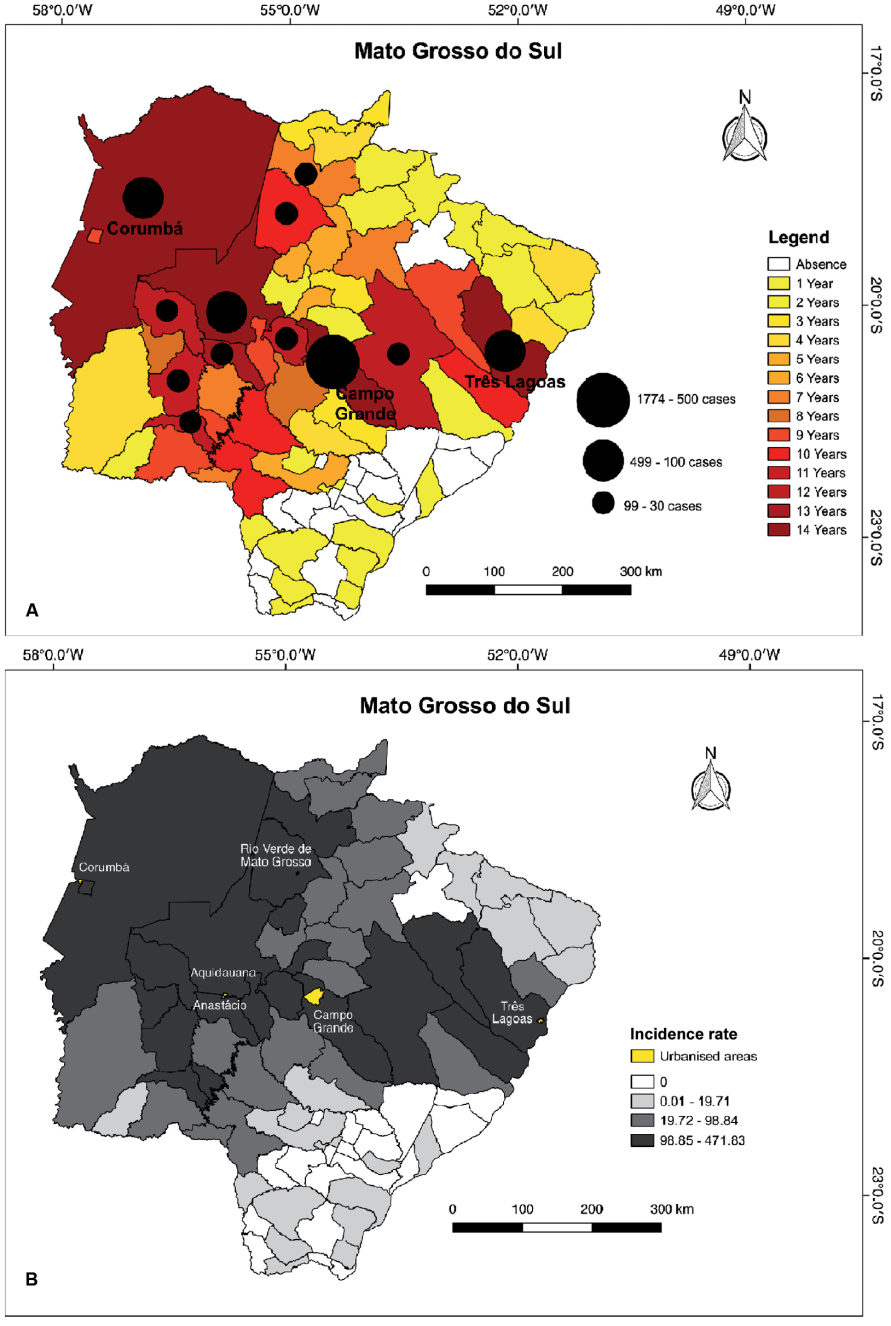
spatial distribution of visceral leishmaniasis cases in Mato Grosso do Sul, 2002-2015. (A) The number of cases (black circles) and the number of years in which these cases were recorded for the period. (B) Classification by incidence rate.

During the period of analysis (2002-2015), 3,137 cases of VL were recorded in 59 municipalities. We observed that the highest incidences were concentrated in the central region of the state, from east to west, passing through Corumbá, Campo Grande, and Três Lagoas (Fig. 2). The highest number of VL cases was recorded in Campo Grande [1,774 (56%)], followed by Três Lagoas [418 (13%)] and Aquidauana [129 (4%)] (Fig. 2A). However, when we analysed the incidence of VL, the highest incidence was noted in Três Lagoas (471.83 cases/100,000 inhabitants), followed by Rio Verde de Mato Grosso (418.17/100,000 inhabitants) and Anastácio (408.48/100,000 inhabitants) (Fig. 2B).

Table shows the annual VL distribution in MS (2002-2015), ENSO variations, and *El Niño* and *La Niña* years. The highest numbers of VL were reported in 2012 (12.37 cases/100,000 inhabitants), 2011 (11.02 cases/100,000 inhabitants), and 2008 (10.83 cases/100,000 inhabitants). The lowest number of VL cases was reported in 2015, with 5.09 cases/100,000 inhabitants.

**TABLE:** Number of cases and incidence of human visceral leishmaniasis (VL) in Mato Grosso do Sul, annual means of El Niño-Southern Oscillation (ENSO) variation (*Niño* 3.4) and classification of *El Niño*, Neutral and *La Niña* phase 2002-2015

Year	Cases	Incidence (cases /100,000hab)	ENSO	Phase
2002	182	8.50	0.59	*El Niño*
2003	193	8.90	0.24	Neutro
2004	238	10.67	0.37	*El Niño*
2005	243	10.73	0.04	Neutro
2006	242	10.53	0.09	Neutro
2007	233	9.99	-0.56	Neutro
2008	253	10.83	-0.71	*La Niña*
2009	197	8.35	0.35	*El Niño*
2010	217	8.86	-0.43	Neutro
2011	273	11.02	-0.85	*La Niña*
2012	310	12.37	-0.03	*La Niña*
2013	244	9.43	-0.22	Neutro
2014	177	6.76	0.25	Neutro
2015	135	5.09	1.47	*El Niño*

**Fig. 3:**
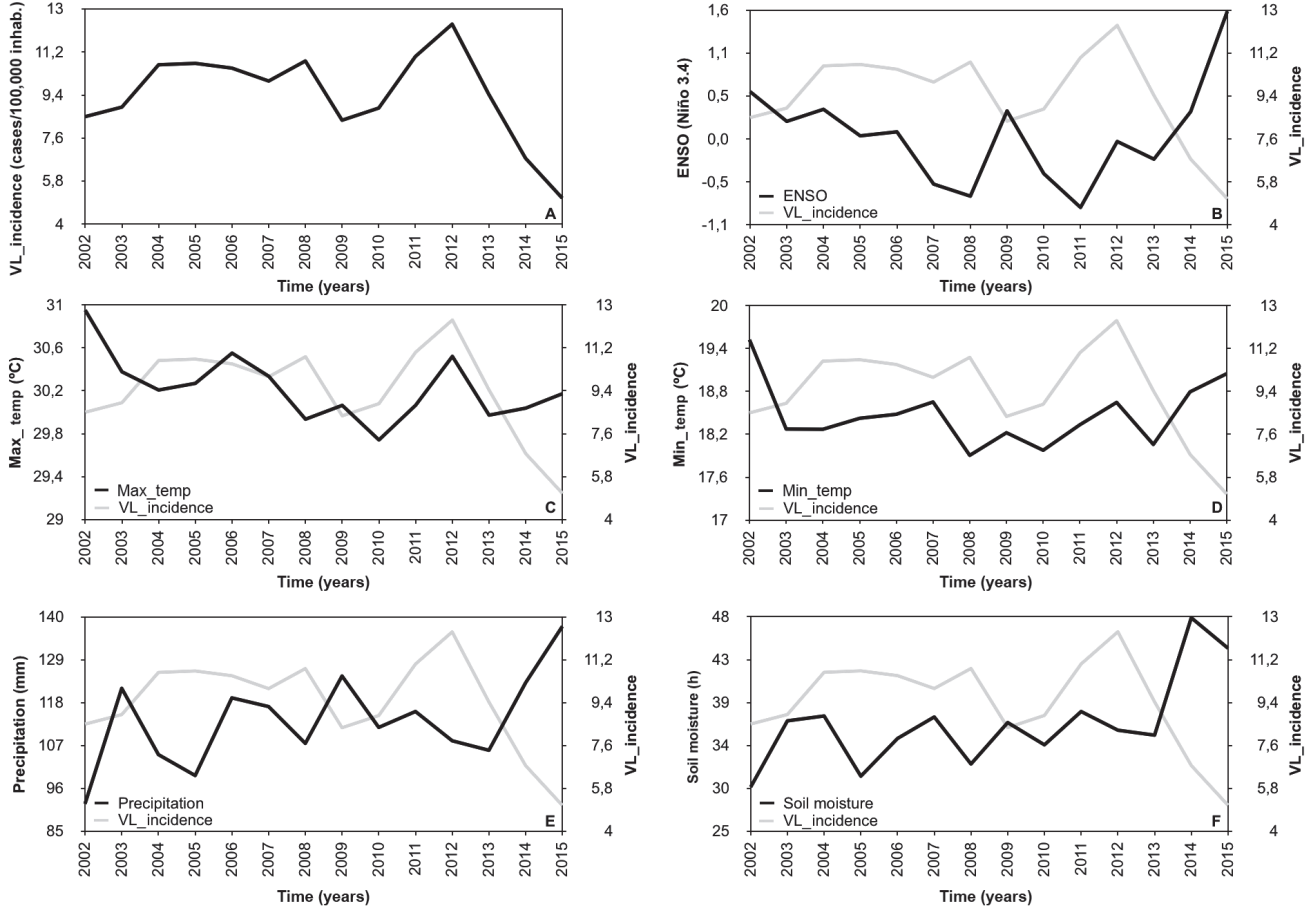
boxplot for the monthly distribution of the incidence of human visceral leishmaniasis in Mato Grosso do Sul, 2002-2015.

Fig. 3 shows the seasonal distribution of VL. The disease was recorded in all months during the 14-year analysis, with an average annual incidence of 0.899/100,000 inhabitants in January and a minimum of 0.725/100,000 inhabitants in June. Considering that the distribution of monthly incidence values does not have symmetrical distribution, in this case, the median represents the best measure of central tendency. Therefore, the months with the highest and lowest median incidence were March (0.864/100,000 inhabitants) and December (0.690/100,000 inhabitants), respectively. Generally, all the analysed variables presented oscillation during the evaluation period.

**Fig. 4:**
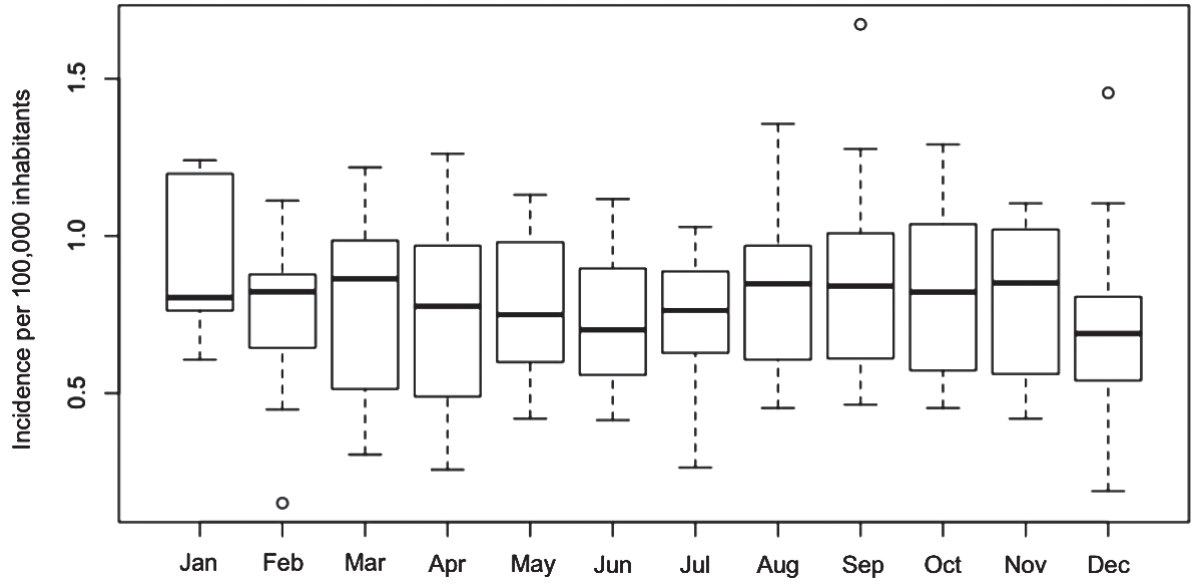
time series of Mato Grosso do Sul, Brazil (2002-2015). (A) Human visceral leishmaniasis (VL) incidence (cases/100,000 inhabitants). (B-F) Climatic variables and human VL incidence. (B) Niño 3.4 variation (El Niño-Southern Oscillation). (C) Maximum temperature. (D) Minimum temperature. (E) Rainfall. (F) Soil moisture.

Fig. 4 shows the VL annual distribution and climatic series for MS from 2002 to 2015. Fig. 4A shows the annual incidence of VL (cases/100,000 inhabitants). Fig. 4B shows the ENSO variation using the Niño 3.4 index. Fig. 4C shows the maximum temperatures with the highest and lowest annual averages recorded in 2002 and 2010, respectively. Fig. 4D shows that that the highest and lowest annual averages for the minimum temperature were recorded in 2002 and 2008, respectively. Fig. 4E shows that the minimum and maximum precipitations were recorded in 2002 and 2015, respectively, and Fig. 4F shows that the minimum and maximum values of soil moisture were recorded in 2002 and 2014, respectively.

**Fig. 5:**
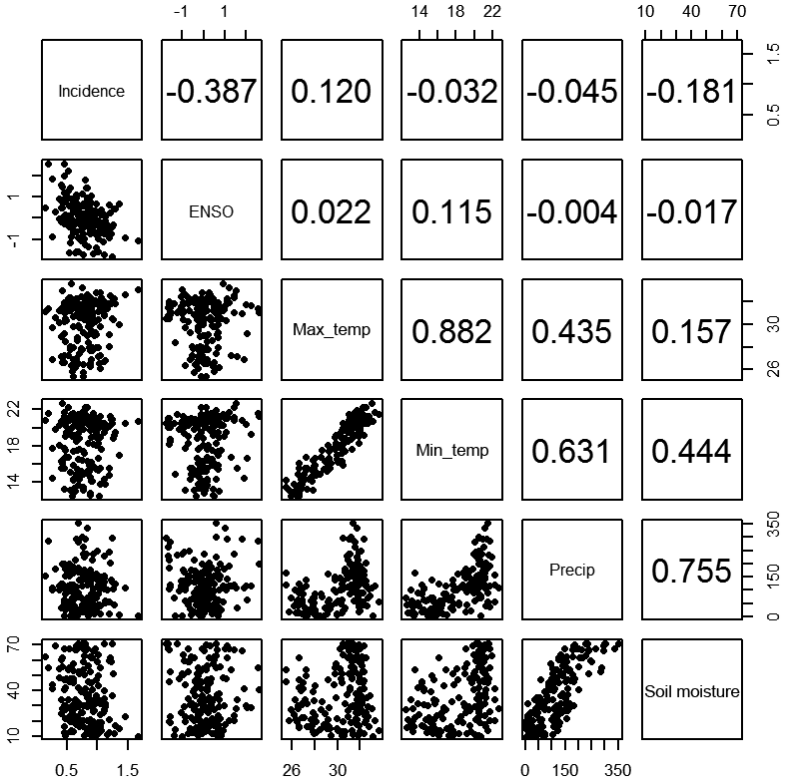
dispersion and correlation matrix between the incidence of human visceral leishmaniasis in Mato Grosso do Sul and El Niño-Southern Oscillation variables — maximum temperature (Max_temp), minimum temperature (Min_temp), precipitation (Precip), and soil moisture — 2002 to 2015. Numbers inside the boxes refer to r value.

Fig. 5 present the dispersion diagrams and correlation coefficients for the variables considered in our study. Within the variables analysed, only the incidence of VL had normal distribution, while all the climatic variables had non-normal distribution. Therefore, all analyses were performed based on Spearman correlation. The results obtained revealed that the annual incidence of VL was negatively associated with the mean ENSO variation (*Niño* 3.4) (r = -0.391, p < 0.001). When an increase in the ENSO mean was observed, a decrease in the incidence of VL was noted. Therefore, it can be observed that in the years with the highest VL incidence (2008, 2011, and 2012), a negative oscillation for ENSO, which was consistent with *La Niña* years, was recorded. While in the years with high ENSO averages, the incidence of VL decreased (5.09/100,000 inhabitants), specifically in 2015, the year with the highest mean value (ENSO = 1.47), which was consistent with *El Niño* years.

A significant negative association was also observed between the incidence of VL and soil moisture (r = -0.181, p = 0.018), indicating the possibility of decreasing the parasitic records in periods with more soaked soil (Fig. 4F). The statistical results of the other variables (i.e. the maximum and minimum temperatures and precipitation) were insignificant (Fig. 4C-E). However, although the linear association between the incidence of VL and precipitation was insignificant (r = -0.045, p = 0.564), a strong association was observed between the variables precipitation and soil moisture (r = 0.755, p < 0.001), indicating that humidity could directly influences precipitation, which is associated with the incidence of VL (Fig. 5). Based on the mean rainfall (Fig. 4E), a low incidence of VL during 2009, 2014, and 2015, years with high rainfall records and positive ENSO means, was also observed.

## DISCUSSION

It seems that the spatial distribution pattern of VL cases in MS is concentrated in the same areas described by Antonialli et al.^10^ from 1993 to 2004, with the highest incidence being reported on the east-west region of the state, from Corumbá to Três Lagoas. It can be noted that 20 municipalities (19 of them are located in the southern region of MS) had no available records of human VL cases from 2002 to 2015, indicating the probability that these areas do not present favorable conditions for VL development. Hence, future studies regarding sandfly diversity and parasite reservoirs and further investigation on the possible underreported and asymptomatic human cases are required to confirm this hypothesis.

Despite the broad territory of the state and the epidemiological complexity of VL, our results demonstrated that the interannual cycles of VL incidence are possibly associated with the ENSO phenomenon. Although the *La Niña* effects on the state are insignificant in MS,^15^ the association between infectious and parasitic diseases and ENSO has already been demonstrated in other countries. A study conducted in Panama indicated an increase in the occurrence of cutaneous leishmaniasis (CL) during the ENSO cold phase (*La Niña*).^16^ In Venezuela, between 1994 and 2003, a significant negative association between CL cases and ENSO was also observed. A significant number of CL cases were reported during the cold phase/*La Niña*; moreover, in that study, the authors observed that during the years where higher ENSO variation was observed, the incidence of CL was lower.^17^ These studies are consistent with the result of our study regarding the association between the ENSO variation and the incidence of VL.

One of the probable factors involved in the variation of the number of VL cases in the present study is the fact that sandfly vectors are influenced by environmental variables, such as temperature, humidity, luminosity, altitude, and vegetation cover.^18^ Thus, these factors may also have an influence on the transmission of the parasite and consequently on VL development.

According to Marcuzzo and Oliveira,^15^ *El Niño* events in MS result in increased maximum daily precipitation, while the *La Niña* phenomena have little influence on the rainfall regime, which remains normal during the cold phase. Therefore, the reduction of VL cases in the state consistent with the occurrence of the *El Niño* phase may be due to a large volume of rainfall in a short period, resulting in soaked soil. Excessive moisture is a limiting factor for the evolution of immature stages (larvae and pupae) of sandflies that develop in the soil.^4^ Although the incidence of VL showed no significant association with precipitation, it presented a negative association with soil moisture. Furthermore, soil moisture values showed a strong association with precipitation (r = 0,755, p < 0,001). It is worth mentioning that the lower incidences of VL were recorded during the years 2009, 2014, and 2015, where higher precipitation volumes were recorded. Therefore, it is believed that during the years with high precipitation and higher soil moisture, reduced VL cases are observed.

Queiroz et al.^5^ cited that an above-average rainfall can reduce the organic matter available in the soil, preventing the immature sandflies’ development. Roger et al.^9^ have shown, in French Guiana, that the mean annual number of cases of leishmaniasis (VL and CL) has a negative association with rainfall. Thus, during the *El Niño* years, which result in high maximum daily precipitation in MS, limited immature sandfly development was observed. However, during the neutral and *La Niña* years, with normal rainfall, the ‘ideal’ moisture favoured vector development, enhancing vector transmission and consequently increasing VL cases.

On the contrary, in some regions worldwide, different associations between some diseases and the ENSO phenomenon are observed. In the region known as the Horn of Africa (Djibouti, Ethiopia, Eritrea, Somalia), *El Niñ*o events increase the risk of Rift Valley fever, cholera, and malaria, while *La Niña* increases the risk of dengue fever, chikungunya, and yellow fever.^19^ However, it is worth emphasising that the vectors of these agents have different epidemiological profiles to that of sandflies.

In the Northeast region of Brazil, studies indicate that increased precipitation influences vector development. In that region, during or shortly after rainy periods, an increase in the number of VL cases and an increase in the population density of sandflies are observed.^20^ The climatic differences between the northeast and central-west regions of Brazil might be the reason for this discrepancy between the results of the references above and the results in the present study. In the northeast, where the average temperature is normally high, *El Niño* results in drought and even higher temperatures. However, with the occurrence of *La Niña*, there is a decrease in this average temperature, enhancing vector development. In MS, the average rainfall and milder temperatures are considered the ideal conditions for sandflies’ dissemination.

Considering that the Pacific Ocean is the largest body of water on Earth, variations in its surface temperature are indicative of an increase or decrease in the planet’s thermal energy.^8^ Thus, during *El Niño*, positive variations may indicate increased global temperature. Although there is no statistical evidence in the present study that confirms the association between the maximum and/or minimum temperatures and the incidence of VL in the state, a negative association between the incidence of VL and ENSO was observed, which is in part an indirect measure of temperature. These results may be due to the temperature variables used, which were obtained based on the total MS area, including forest regions (Cerrado, Pantanal, and Atlantic Forest). Therefore, it is possible that when analysing only the urbanised areas, where human infections are likely to occur, the temperature-VL association might be confirmed.

Temperatures up to 30ºC increase the reproduction of the vector insects - mosquitoes^19^ and sandflies^21^ ^)^ - and decrease the maturation time of the pathogen in the vector. However, temperatures above this threshold may reduce insect survival due to fragility to desiccation. Females are significantly influenced by climatic variations, affecting their oviposition ability when temperatures are higher and humidity levels are lower.^6^ Regarding the biology of *Lu. longipalpis*, a vector of *L. infantum*, Guzman and Tesh^22^ have shown that temperatures approximately 18ºC negatively influence the immature forms (larvae and pupae) of sandflies, but the adult sandflies’ lifespan is higher. Thus, the exposure of individuals - hosts and reservoir - to the vectors can be increased. Rivas et al.^23^ showed that at 30ºC, the maximum peak of the abundance of *Lu. longipalpis* was considered lower than the other lower temperature regimes (20ºC and 25ºC). The studies cited above indicate that lower temperatures seem to favour sandfly reproduction and, consequently, lead to an increase in the number of VL cases. However, high temperatures, related to *El Niño* phases, do not favour sandfly reproduction, negatively affecting vector development. However, it is worth emphasising that there must be a threshold in the sandfly species for both low and high temperatures that either influences or not the survival of adult insects and the development of immature sandflies.

Murdock, Sternberg, and Thomas^24^ have shown that even small changes in temperature can affect the dynamics of malaria transmission, indicating that an increment in temperature can reduce mosquitoes’ vector capacity by up to 89%. A similar pattern seems to influence sandflies as there is a decrease in the abundance of the species due to an increase in temperature.^16^ Moreover, digestion, metabolic processes, and the development of different species of sandflies are significantly affected by ambient temperature.^25^

Moreover, the development of a parasite in a vector is also influenced by environmental conditions. Hlavacoca, Votypka, and Volf^26^ showed that for the species *Lu. longipalpis*, temperatures lower than 20ºC allow the development, although slower, of the protozoan *Leishmania peruviana*. However, at 26°C, the infection rate and parasite load were significantly low. The authors suggest that lower temperatures delay the growth of the parasite after the blood meal; however, there is no impairment in its development in the sandfly’s gut. A similar result was observed for *Phlebotomus papatasi*, another sandfly species. The insects kept at 23ºC showed a higher rate of infection by *Leishmania major* compared to the dipterans kept at 28ºC. Moreover, lower temperatures increase the adult sandflies’ lifespan and prolong their digestion, allowing greater time for the development of infection.^25^ These facts support the hypothesis that events such as *El Niño*, with higher temperatures and rainfall, are possibly associated with a decrease in the incidence of VL in MS, while its opposite events, *La Niña* and neutral phases, which have no influence on the climate in the MS, are associated with the increase in the occurrence of VL cases in the present study.

Considering climate changes will expectedly occur more frequently in the future, specifically the increase in global temperature, some authors suggest that the pattern of distribution of different diseases, such as VL, would possibly change.^7^ In temperate areas, such as the regions in Europe and North America, an increase in number of vectors and VL cases are reported, confirming that the distribution pattern of the parasite and its respective vectors has changed.^1^ ^,^ ^27^ ^,^ ^28^

Mathematical prediction indicates that with increasing global temperature, specifically in the tropical region, it is expected that there will be a decrease in the incidence rates of VL in this region since the vector insects would migrate to areas with higher altitude.^28^ ^,^ ^29^ Similar results were observed by Escobar et al.^7^ in Ecuador, where the distribution of different insect vectors of arbovirus, leishmaniasis, malaria, and Chagas disease agents could be altered with the increase of atmospheric temperature. In this context, some authors stated that the increase in global temperature tends to reduce the risk of vector-borne diseases, counteracting the negative effects of climate changes commonly associated with human health.^7^ ^,^ ^29^

Finally, based on the monthly distribution of the incidence of VL, VL cases in all months of the 14-year analysis were recorded, consistent with the results of several studies regarding vectors observed in MS. The occurrence of *Lu. longipalpis* and *Lu. cruzi* in all months of the year in different regions of the state may explain the continuity of outbreaks of the disease during the year. Therefore, the presence of the vector can be used as a substitute to explain the occurrence of the disease through the annual course.^5^ ^,^ ^18^

The variables studied in this research represent only a part of the factors that may influence the incidence of VL in MS. For example, deforestation and socioeconomic factors were not taken into consideration. Thus, further studies are required to evaluate the association between VL and different variables to better understand the aspects involved in the dynamics of the disease in the state. Environmental changes, urban migrations, basic sanitation, and the role of the reservoir in various clinical conditions are some of the factors that can facilitate the urbanisation of the VL. Failures regarding the notification of cases may also influence the number of cases of the disease in the state.^30^ More sophisticated analytical methods incorporating temporal and spatial components simultaneously, such as regression models, can improve the understanding of the interrelationship between the links of the epidemiological chain. However, the use of these approaches depends on the availability and quality of the data.

One of the possible limitations in the present study was as follows: only the urbanised areas, where most of the VL cases are concentrated, were taken into consideration. The regional climatic variables were obtained for the MS region as a whole. Large forest areas, such as the Pantanal, may influence the values obtained for temperature (maximum and minimum), rainfall, and humidity. Thus, the most local analyses should be ideally obtained, contributing to the results presented here. Furthermore, studies assessing the association between ENSO and climate change in MS are emerging, and only one reference was found,^15^ indicating the possible effects of *El Niño* and *La Niña* events in the state.

In summary, the frequent occurrence of extreme climatic phenomena, such as *El Niño* and *La Niña* phases, may significantly influence the incidence of VL. *El Niño* reduces the incidence of VL and decreases the possible emergence of new VL cases probably due to the increase of rainfall volume and humidity affecting the vector’s biology. The possible effects of *La Niña* and the higher incidences of VL observed during these events need to be better evaluated to explain the parasite’s cycle in MS.
